# A prospective cross-sectional study of tuberculosis in elderly Hispanics reveals that BCG vaccination at birth is protective whereas diabetes is not a risk factor

**DOI:** 10.1371/journal.pone.0255194

**Published:** 2021-07-29

**Authors:** Julia M. Scordo, Génesis P. Aguillón-Durán, Doris Ayala, Ana Paulina Quirino-Cerrillo, Eminé Rodríguez-Reyna, Francisco Mora-Guzmán, Jose A. Caso, Eder Ledezma-Campos, Larry S. Schlesinger, Jordi B. Torrelles, Joanne Turner, Blanca I. Restrepo

**Affiliations:** 1 Host Pathogen Interactions and Population Health Programs, Texas Biomedical Research Institute, San Antonio, TX, United States of America; 2 The University of Texas Health Science Center of San Antonio, San Antonio, TX, United States of America; 3 Secretaria de Salud de Tamaulipas, Reynosa, Matamoros and Ciudad Victoria, Tamaulipas, México; 4 University of Texas Health Science Center at Houston, School of Public Health, Brownsville, TX, United States of America; 5 Biology Department, University of Texas Rio Grande Valley, Edinburg, TX, United States of America; 6 School of Medicine, South Texas Diabetes and Obesity Institute, University of Texas Rio Grande Valley, Edinburg, TX, United States of America; The University of Georgia, UNITED STATES

## Abstract

**Background:**

Aging increases the risk of tuberculosis (TB) and its adverse outcomes, but most studies are based on secondary analyses, and few are in Hispanics. Diabetes is a risk factor for TB in adults, but its contribution in the elderly is unknown. We aimed to identify the role of diabetes and other risk factors for TB in elderly Hispanics.

**Methods:**

Cross-sectional study among newly-diagnosed TB patients, recent contacts (ReC), or community controls (CoC) totaling 646 participants, including 183 elderly (>60 years; 43 TB, 80 ReC, 60 CoC) and 463 adults (18 to 50 years; 80 TB, 301 ReC and 82 CoC). Host characteristics associated with TB and latent *Mycobacterium tuberculosis* infection (LTBI) were identified in the elderly by univariable and confirmed by multivariable logistic regression.

**Results:**

LTBI was more prevalent among the elderly CoC (55% *vs*. 23.2% in adults; p<0.001), but not in ReC (elderly 71.3% *vs*. adult 63.8%); p = 0.213). Risk factors for TB in the elderly included male sex (adj-OR 4.33, 95% CI 1.76, 10.65), smoking (adj-OR 2.55, 95% CI 1.01, 6.45) and low BMI (adj-OR 12.34, 95% CI 4.44, 34.33). Unexpectedly, type 2 diabetes was not associated with TB despite its high prevalence (adj-OR 0.38, 95% CI 0.06, 2.38), and BCG vaccination at birth was protective (adj-OR 0.16, 95% CI 0.06, 0.45).

**Conclusions:**

We report novel distinctions in TB risk factors in the elderly *vs*. adults, notably in diabetes and BCG vaccination at birth. Further studies are warranted to address disparities in this vulnerable, understudied population.

## Introduction

Aging is associated with immune function decline consequent to cellular immunosenescence and inflammaging, and is identified as a risk factor for respiratory tract infections such as tuberculosis (TB) [1, 2]. TB caused an estimated 1.4 million deaths and 10 million new cases in 2019. Approximately one-fourth of the world’s population has latent *Mycobacterium tuberculosis* infection (LTBI) [3]. The relative risk of TB in the elderly is 1.5-fold higher than in adults (21 to 64 years old), and the risk of mortality is ≥ 5-fold higher, with rates ranging from 20 to 30% [4–6]. Elderly patients are difficult to diagnose due to the lack of classical symptoms and to impaired or reduced responses to diagnostics such as the tuberculin skin test. This leads to frequent delays in treatment initiation and in some cases to post-mortem diagnosis [7–9].

Most studies in older people with TB are based on secondary analysis of data [10–12]. These studies have identified risk factors for TB in the elderly, including aging itself, male sex, smoking, and malnutrition, undernutrition or low body mass index (BMI) [11]. Although diabetes increases the risk of TB by 3-fold among adults [13], the link between TB and diabetes in the elderly has not been formally studied. Instead, association studies among all adults suggest a stronger link to diabetes among middle-aged *vs*. older adults (e.g. ≥ 60 years) [14–17]. Our prior studies among Hispanics in the states of Tamaulipas (Mexico) and Texas (US) identified diabetes as a major contributor to TB, with a 25% population attributable risk fraction [17], but elderly individuals were mostly excluded [18].

Here we sought to identify risk factors for LTBI or TB among the elderly in a Hispanic community across the US-Mexico border [19]. We conducted a cross-sectional study with enrollment of newly-diagnosed TB patients, recent contacts (ReC), and community controls (CoC) among elderly individuals (>60 years), and used adults (18 to 50 years old) as reference. We found shared risk factors for TB between the elderly and adult populations, but most importantly, we identified unique aspects in the elderly, most notably the lack of association between TB and diabetes and a potential protective role of BCG vaccination at birth.

## Methods

### Ethical statement

This study was approved by the institutional review boards in Mexico (SST/SCAME/DCES/597/2017, Secretaría de Salud de Tamaulipas) and the United States (HSC-SPH-17-0990, University of Texas Health Houston), and all participants signed an informed consent.

### Participant enrollment and characteristics

**Fig 1:**
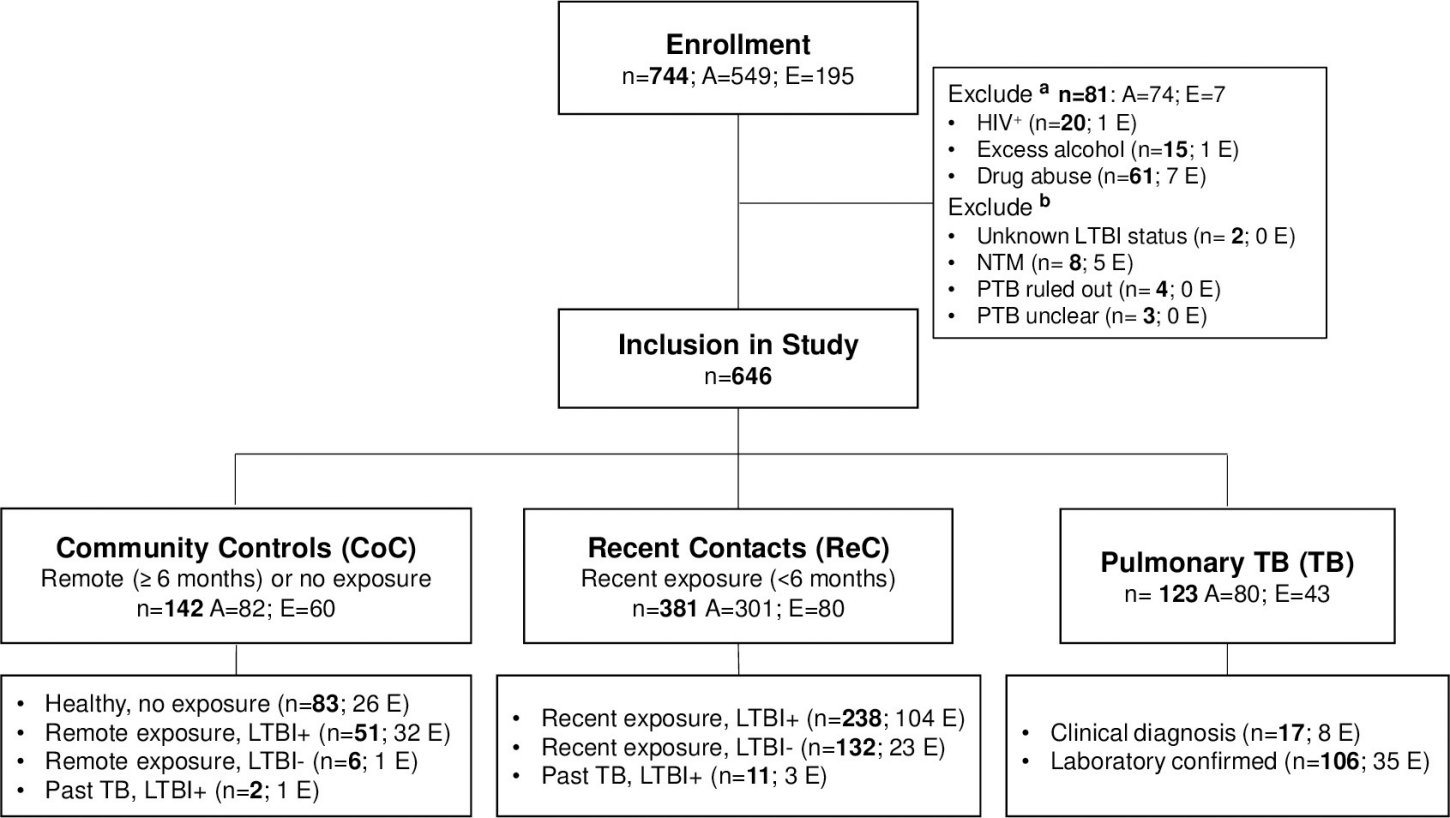
Enrollment algorithm. Adults (A) and elderly (E) individuals identified in the community (Community controls, CoC), or in pulmonary clinics as newly-diagnosed pulmonary TB patients (TB), or contacts of TB cases with reported exposure ≤6 months before enrollment (Recent contacts, ReC), were invited to participate. TB diagnosis was based on laboratory findings (positive culture and/or acid-fast sputum smear) or clinical criteria based on physician diagnosis and abnormal chest X-ray. LTBI, Latent TB infection. ^**a**^Exclusion based on HIV infection, excessive alcohol use, and drug abuse, with some having more than one of these conditions. ^**b**^Exclusion due to unknown LTBI status, non-tuberculosis mycobacteria (NTM) or pulmonary TB diagnosis ruled out/unclear.

**Fig 2:**
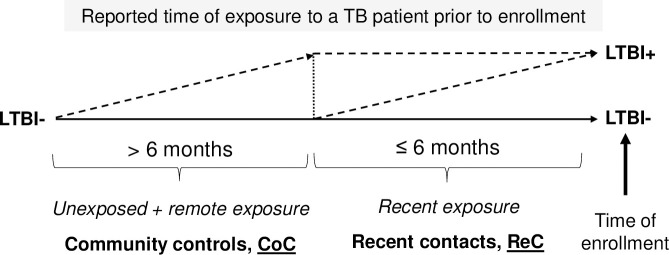
Definitions for community controls and recent contacts. Community controls were identified in the community (not in pulmonary clinics) and reported no previous exposure or a remote exposure to a pulmonary TB patient >6 months before enrollment. Recent contacts reported exposure to an active TB case ≤6 months before the time of enrollment. Community controls and recent contacts with at least one positive IGRA assay were classified as LTBI+, and when both negative, then as LTBI-.

Adults (18 to 50 years) and elderly (>60 years) participants were enrolled at pulmonary and community clinics in South Texas and northern Tamaulipas, Mexico, between 2017 and 2020. Participants included newly-diagnosed active pulmonary TB patients enrolled prior to or within one week of TB treatment (TB), recent TB contacts (ReC), and community controls (CoC) (Fig 1). TB diagnosis was based on isolation of *M*. *tuberculosis* or positive sputum smear (laboratory diagnosis), or by clinical symptoms consistent with pulmonary TB and an abnormal chest X-ray (clinical TB) [20]. ReC were defined by exposure for ≥ 5 h to an active TB case ≤ 6 months prior to enrollment. CoC were enrolled in the community and reported no previous exposure or a remote exposure > 6 months before enrollment (Fig 2). LTBI was assessed by Interferon Gamma Release Assays (IGRA) [QuantiFERON-Gold-in-tube or QuantiFERON-plus (Qiagen, Germantown, MD) or T-SPOT.TB (Oxford Immunotec, Oxford)]. Socio-demographic information was recorded at enrollment as described previously [21]. Briefly, macrovascular and microvascular diseases, use of diabetes medications in the past month, or taking non-steroidal anti-inflammatory drugs (NSAIDs) in the past week was based on self-reporting. Excessive alcohol intake was based on a validated questionnaire [22]. Drug abuse was self-reported as daily or weekly use of recreational injectable or non-injectable drugs. Diabetes was based on hyperglycemia (fasting ≥126 mg/dL in most cases, or random ≥200 mg/dL), chronic hyperglycemia (HbA1c ≥6.5%), or self-reported diagnosis [23]. Prediabetes was based on HbA1c between 5.7% and 6.4% or fasting glucose between 110 and 125 mg/dL. HIV infection was based on self-reporting in non-TB cases, or blood test confirmed in TB patients. Total cholesterol, HDL cholesterol and triglycerides were determined using Lipid Panel Test Strips (PTS Diagnostics), and low-density cholesterol (cLDL) was calculated [24].

### Data analysis

The study design followed the STROBE guidelines [25]. Data were entered into Microsoft Access and exported into SAS version 9.4 (Cary, NC) for quality control and analysis. Additional analyses were performed using GraphPad Prism version 9.0 (La Jolla, CA). Categorical variables were compared with the Chi-square or Fisher’s exact test. Continuous variables were analyzed for differences in medians using Wilcoxon test or Kruskal-Wallis with Dunn’s multiple comparisons test for > 2 groups. For multivariable logistic regression models, variables were selected based on a *p* value ≤ 0.099 or biological relevance. Given the low incidence of TB in our study population, we report odds ratios and refer to them as risk factors given their close approximation to risk ratios [17]. Significance was set as *p* ≤ 0.050 and marginal significance at *p* ≤ 0.099.

## Results

### Participant enrollment and characteristics

A total of 744 participants were enrolled (549 adults and 195 elderly). Adults that had a higher prevalence of HIV, excessive alcohol consumption or drug abuse, were excluded given our primary interest in identifying risk factors for TB in the elderly (Fig 1). We further excluded TB suspects with non-tuberculous mycobacteria, unclear or ruled out TB, or ReC with unknown LTBI status. The remaining 646 participants comprised 123 new-diagnosed pulmonary TB (43 elderly and 80 adults), 381 ReC (80 elderly and 301 adults), and 142 CoC (60 elderly and 82 adults; Fig 1). More than half of the participants were females (62.8%) and 72.9% married. Only 39% had an education beyond middle school, 74.8% had health insurance, 71.2% were non-smokers with only 12.6% current smokers. Two-thirds were obese or overweight (66.1%), 71.9% had central obesity, and 28.9% had diabetes (98% type 2 diabetes). Most were BCG vaccinated (88.5%) at birth, and only 2.5% had a history of TB (Table 1).

**Table 1:** Characteristics of the elderly *vs*. adults, by TB study groups.

	All		Non-TB					TB				
			Adults		Elderly			Adults		Elderly		
	n	% or	n	% or	n	% or median (IQR)	p value	n	% or median (IQR)	n	% or median (IQR)	p value
		median (IQR)		median (IQR)								
**Sociodemographics**												
**Age, in years**	646	43.0 (28.0)	383	38 (14)	140	67 (10.5)	**<0.001**	80	37.0 (21.0)	43	68.0 (11.0)	**<0.001**
**Male sex**	240	37.2%	126	32.9%	38	27.1%	0.209	45	56.3%	31	72.1%	**0.085**
**Marital status**							**<0.001**					**0.003**
Never married	104	16.1%	68	17.8%	7	5.0%		21	26.3%	8	18.6%	
Ever married *	471	72.9%	306	79.9%	84	60.0%		56	70.0%	25	58.1%	
Widowed	71	11.0%	9	2.3%	49	35.0%		3	3.8%	10	23.3%	
**Smoking**							0.716					**0.099**
Never	459	71.2%	284	74.2%	106	75.7%		49	62.0%	20	46.5%	
Past or Current	186	28.8%	99	25.8%	34	24.3%		30	38.0%	23	53.5%	
**Smoking**—**pack per year**	644	0.01 (0.40)	383	0 (0.1)	140	0 (0.0)	0.697	79	0.01 (1.50)	42	0.55 (5.10)	**0.017**
**Socioeconomic indicators**												
**Highest education**							**<0.001**					**<0.001**
Up to Middle School	394	61.0%	197	51.4%	113	80.7%		46	57.5%	38	88.4%	
High School or College	252	39.0%	186	48.6%	27	19.3%		34	42.5%	5	11.6%	
**Health insurance**	482	74.8%	269	70.2%	116	82.9%	**0.004**	66	82.5%	31	73.8%	0.259
**Household family size**	566	4.0 (2.0)	331	4.0 (2.0)	119	3.0 (3.0)	**<0.001**	81	4.0 (2.0)	38	3.0 (4.0)	0.228
**Health conditions**												
**Diabetes (2 categories)**	187	28.9%	69	18.0%	66	47.1%	**<0.001**	31	38.8%	21	48.8%	0.280
**Diabetes (3 categories)**							**<0.001**					**<0.001**
No diabetes	311	48.1%	233	60.8%	32	22.9%		39	48.8%	7	16.3%	
Pre-diabetes	148	22.9%	81	21.1%	42	30.0%		10	12.5%	15	34.9%	
Diabetes	187	28.9%	69	18.0%	66	47.1%		31	38.8%	21	48.8%	
**Obesity, BMI**	646	27.2 (7.5)	383	28.6 (7.5)	140	27.8 (6.9)	0.266	80	22.2 (7.0)	43	21.7 (5.5)	0.443
**Obesity, BMI categories**							0.148					0.125
Under/Normal (<24.9)	219	33.9%	86	22.5%	40	28.6%		57	71.3%	36	83.7%	
Over/Obese (≥25)	427	66.1%	297	77.5%	100	71.4%		23	28.8%	7	16.3%	
**WHR** (M ≥ 0.90; F ≥0.86)	454	71.9%	284	74.2%	111	79.3%	0.399	37	47.4%	22	52.4%	**0.078**
**Macrovascular diseases**	153	23.7%	51	13.3%	80	57.1%	**<0.001**	7	8.4%	15	34.9%	**<0.001**
**Microvascular diseases**	169	26.2%	68	17.8%	65	46.4%	**<0.001**	20	24.1%	18	41.9%	**0.040**
**NSAIDs in past week**	128	23.3%	48	12.5%	40	28.6%	**<0.001**	24	31.2%	16	42.1%	0.247
**TB-related conditions**												
**Past TB**	13	2.5%	9	2.3%	4	2.9%	0.742	10	12.1%	4	9.3%	0.642
**Latent TB infection** **	211	55.1%	212	55.4%	91	65.0%	**0.048**		N/A		N/A	
**BCG vaccine**	570	88.5%	341	89.0%	131	93.6%	0.158	70	87.5%	28	65.1%	**0.003**

Data expressed as column % for categorical variables or median (interquartile range, IQR) for continuous; Normal range values for each parameter shown in parenthesis

* Ever married includes married, cohabitation, divorced or separated; NSAIDs, non-steroidal anti-inflammatory drugs; WHR, waist-hip ratio or Central obesity; M, Male; F, Female; p values ≤ 0.099 shown in bold

** Latent TB infection based on a positive T-Spot.TB or QuantiFERON assay, LTBI only evaluated in non-TB study groups; BMI, Body-mass index

### Age-associated characteristics amongst non-TB or TB participants

We first sought to identify host characteristics that distinguished elderly *vs*. adult groups, given their higher risk of active TB, or adverse TB outcomes [11, 12, 26]. When analyzing the non-TB groups (CoC and ReC groups), we found that most of the characteristics that distinguished the elderly from adults were similar (S1 Table). Therefore, for most analysis we merged both groups into one ‘non-TB category’. An exception was the analysis related to LTBI given its higher prevalence in the elderly *vs*. adults among CoCs (E 55.0% *vs*. A 23.2%; p <0.001), but similarity in ReCs (E 64.1% *vs*. A 71.3%; p 0.233; S1 Fig; S1 Table).

Among the non-TB participants, the following features distinguished the elderly *vs*. adults (Table 1; S1 and S2 Figs). Regarding socio-demographics, the elderly had differences in marital status (mostly widowed; p <0.001) and socioeconomic indicators [lower education (p <0.001) and smaller family size (p <0.001), but higher frequency of health insurance (p = 0.004)]. Regarding health conditions, the elderly had a higher prevalence of diabetes and pre-diabetes (p <0.001), LTBI (p 0.048), macrovascular and microvascular diseases (p <0.001), and use of anti-inflammatory medications (p <0.001). Glucose-related measurements (Table 2) were higher (hyperglycemia and HbA1c; p <0.001) which is consistent with their diabetes status. Lipid profiles (total, LDL and HDL cholesterols and triglycerides) were also higher in the elderly (Table 2). Regarding hematology indices, the elderly had higher eosinophils (p = 0.045) but lower platelets (p <0.001) and hemoglobin levels (p 0.047; S2 Table).

**Table 2:** Lipid and glucose profiles in elderly *vs*. adults, by TB status.

	Non-TB					TB				
	Adults		Elderly			Adults		Elderly		
	n	%	n	%	p value^a^	n	%	n	%	p value
**Lipid profiles, (mg/dl)**^**b**^										
**High cholesterol** (200)	48	12.6%	29	20.9%	**0.020**	5	6.3%	1	2.3%	0.335
**Low HDL** (40 M, 50 F)	287	75.1%	90	64.7%	**0.019**	58	72.5%	33	76.7%	0.609
**High LDL** (100)	144	38.0%	65	46.8%	**0.072**	15	18.8%	6	14.0%	0.500
**High Triglycerides** (150)	97	25.3%	46	32.9%	**0.087**	6	7.5%	3	7.0%	0.915
**Glucose-related measurements**										
**Glycemia**					**<0.001**					0.153
Normoglycemia	295	78.0%	77	55.4%		45	57.0%	32	74.4%	
Impaired Fasting Glucose	28	7.4%	22	15.8%		8	10.1%	2	4.7%	
Hyperglycemia	55	14.6%	40	28.8%		26	32.9%	9	20.9%	
**Chronic hyperglycemia, HbA1c**					**<0.001**					0.144
Normal (<5.7%)	235	61.4%	36	25.7%		39	48.8%	8	18.6%	
Pre-diabetes (5.7–6.4%)	89	23.2%	56	40.0%		11	13.8%	18	41.9%	
Chronic hyperglycemia (≥ 6.5%)	59	15.4%	48	34.3%		30	37.5%	17	39.5%	

^a^ p values ≤ 0.099 shown in bold

^b^ Normal range values for each parameter shown in parenthesis. Abbreviations: M, Male; F, Female.

Among TB patients, the following characteristics were significantly different between the elderly and adults (Table 1 and S1 Fig). Regarding sociodemographics, there was a higher prevalence of males (p = 0.085), widowed status (p = 0.003) and smoking-packs per year (p = 0.017) in the elderly. Regarding socioeconomics, the elderly had a lower education (p <0.001). Regarding health-related conditions, the elderly had more pre-diabetes or diabetes (p <0.001), macrovascular (p <0.001) and microvascular (p = 0.040) diseases, and lower prevalence of BCG vaccination (p = 0.003). Regarding lipid- and glucose-related measurements, there were no differences (Table 2). Regarding hematology indices, the ratios of neutrophils/lymphocytes and monocytes/lymphocytes were higher in the elderly (S2Table and S2 Fig).

Altogether, we found that known risk factors for TB in adults, such as lower education and dysglycemias, were significantly higher in the elderly *vs*. adults with or without TB [27, 28]. In the elderly, pre-diabetes and diabetes accounted for an overall 76.6% in non-TB and 83.7% in TB cases. Given the high prevalence of dysglycemias, we further examined if these were associated with TB status in our cohort.

### Risk factors for TB in the elderly

In order to determine if diabetes or other host characteristics are risk factors for TB in our elderly Hispanic cohort, we compared the elderly who had TB *vs*. those who did not (S3 Table and S3 Fig). For diabetes and related conditions, the elderly TB participants had lower prevalence of impaired fasting glucose (4.7 *vs*. 15.7%) or hyperglycemia (20.9 *vs*. 28.6%; p = 0.058). Regarding obesity and lipid profiles, the elderly with TB *vs*. no TB, had a higher prevalence of lower BMI and low central obesity (p <0.001), lower total cholesterol (p = 0.004), LDL cholesterol (p <0.001) and triglycerides (p <0.001). Regarding socio-demographics, the elderly with TB had a higher proportion of males or smoking index (p <0.001). The elderly TB participants had a lower prevalence of BCG vaccination (p <0.001) or macrovascular diseases (p = 0.011).

**Fig 3:**
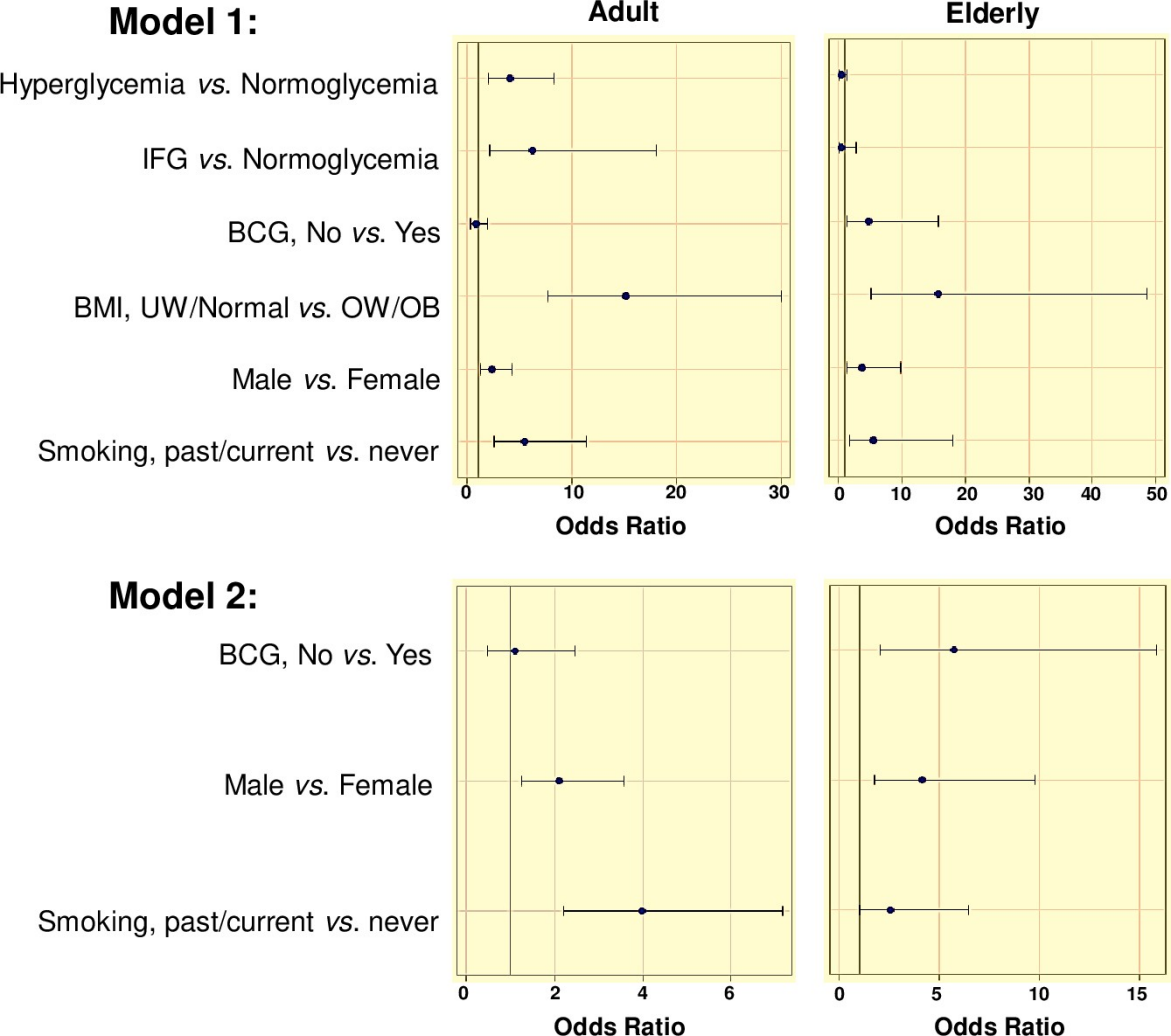
Multivariable logistic regression models of odds of TB vs non-TB among adults or elderly, for different host characteristics. Host characteristics with a *p* value ≤0.09 by univariable analysis were selected as indicated in the text for model 1. For model 2, BMI and glycemia were removed from model 2, given that they may be modified once TB develops. Graphs show odds ratio with Wald 95% CI. Abbreviations: IFG, impaired fasting glucose; UW, underweight (BMI <18.5); OW/OB, overweight/obese (BMI >25); BMI, body-mass index; BCG, Bacillus Calmette-Guérin vaccinated.

To determine if these host characteristics were independently associated with TB in the elderly, we conducted a multivariable logistic regression analysis. In our initial model 1, we evaluated glycemia as a diabetes-defining variable, together with sex, smoking history, BCG vaccination and BMI (selected from the variables defining obesity and lipid profiles). Our analysis showed that hyperglycemia and impaired fasting glucose were not associated, while male sex, BCG vaccination and BMI remained independently associated with TB, and smoking was associated when modeling ‘past’ *vs*. ‘never or current’ TB (Table 3; Fig 3). Given the unexpected association with BCG vaccination, in model 2 we removed BMI and dysglycemia since they could be a consequence of TB. However, absence of BCG vaccination remained associated with risk of TB in the elderly, along with male sex and smoking.

**Table 3:** Univariable and multivariable models for odds of TB *vs*. non-TB patients by host characteristics, in the elderly or adults.

	Elderly: TB vs non-TB			Adults: TB vs non-TB			
	OR (95% CI)	Model 1 ^a^:	Model 2 ^b^:		OR (95% CI)	Model 1:	Model 2:	
			Adj-OR (95% CI)				Adj-OR (95% CI)	
		Adj-OR (95% CI)	Exclude BMI, glycemia			Adj-OR (95% CI)	Exclude BMI, glycemia	
**Male vs female sex**	**6.93 (3.23, 14.87)**	**3.92 (1.41, 10.88)**	**4.33 (1.76, 10.65)**		**2.65 (1.61, 4.37)**	**2.80 (1.49, 5.25)**	**2.63 (1.56, 4.46)**	
**Smoking, ’Past’ vs ’current or never’**	**5.37 (2.46, 11.70)**	**5.43 (1.627, 18.122)**	**2.55 (1.008, 6.449)**		**4.71 (2.68, 8.29)**	**5.41 (2.57, 11.38)**	**3.99 (2.21, 7.19)**	
**BCG vaccine, No vs Yes**	**7.86 (3.13, 19.74)**	**5.36 (1.68, 17.10)**	**6.15 (2.24, 16.92)**		1.17 (0.56, 2.44)	0.73 (0.30, 1.77)	0.98 (0.45, 2.16)	
**Glycemia**								
Normoglycemia	1.0	1.0			1.0	1.0		
Impaired Fasting Glucose	**0.22 (0.05, 0.99)**	0.38 (0.06, 2.38)			2.11 (0.93, 4.76)	**5.84 (2.07, 16.49)**		
Hyperglycemia	0.54 (0.24, 1.25)	0.52 (0.18, 1.46)			**3.30 (1.92, 5.80)**	**4.37 (2.22, 8.58)**		
**Diabetes**	1.07 (0.54, 2.12)				**2.88 (1.71, 4.84)**			
**Obesity**								
Underweight/normal	**12.99 (5.34, 31.58)**	**12.34 (4.44, 34.33)**			**9.01 (5.26, 15.42)**	**14.30 (7.48, 27.32)**		
Overweight/Obese	1.0	1.0			1.0	1.0		

^a^ Model-1. Controlled for all variables in the table.

^b^ Model-2. Model 1, except for BMI and glycemia; 95% CI with p values ≤ 0.099 shown in bold; In sensitivity analysis, additional models were evaluated: Model 1A. When glycemia was replaced by diabetes (2 categories) in Model 1, diabetes remained associated with TB in adults (adj-OR 4.08, 95% CI 2.13, 7.80), but not in the elderly [adj-OR 1.70 (0.65, 4.45)]. Model 1B. Addition of education or health insurance did not modify the associations already observed in Model 1.

Previous studies in adults, including our own, indicated that diabetes is a risk factor for TB, and that BCG vaccination does not confer protection for TB development [17, 29]. To confirm if these findings would hold in our adult cohort, we ran the same multivariable models as described for the elderly. Our findings confirmed that in adults there are higher odds of hyperglycemia (adj-OR 4.37, 95% CI 2.22, 8.58) or impaired fasting glucose (adj-OR 5.84, 95% CI 2.07, 16.49) in TB patients (model 1), and a lack of association between TB and BCG vaccination (models 1 and 2; Table 3 and Fig 3). Past smoking, male sex and lower BMI were also associated with TB in both models (Fig 3).

### Host factors associated with BCG vaccination

Given our observed protective effect of BCG vaccination for TB in the elderly (Fig 3), we evaluated if there was another host characteristic associated with BCG vaccination that we had not identified, that could influence TB risk (e.g. a confounding factor; S4 Table). Among all study participants, BCG vaccination was more prevalent in females *vs*. males (p 0.014), non-TB *vs*. TB participants (p <0.001), individuals with higher education (p = 0.027) and access to health insurance (p 0.027). After controlling for age, sex and TB, BCG vaccination remained associated with higher education level and access to health insurance. Therefore, we expanded the multivariable analysis shown in Fig 3, by adding education or health insurance to the models. However, neither of these variables affected the associations already noted, i.e. risk of TB and lack of BCG vaccination, and the lack of association with diabetes, among the elderly.

### Clinical characteristics of the elderly with TB

Since the diagnosis of TB can be challenging in the elderly [30], we also evaluated whether the elderly differed in their microbiological or clinical presentation of active TB when compared to adults. Our results showed no significant differences in sputum acid-fast smear results or grade, nor the proportion with positive culture (Table 4). Signs and symptoms were similar for cough, productive cough, hemoptysis, chest pain or weight loss, but other important distinguishing features were observed in the elderly. Namely, elderly participants were less likely to report fever or chills, and had a longer history of cough or weight loss prior to TB diagnosis.

**Table 4:** TB diagnostic criteria and signs and symptoms, by age groups.

**Diagnostic criteria**						
	**Adults**	**Elderly**				
	**n (%)**	**n (%)**	**p value**			
**Positive AFB Smear**	75 (91.5%)	39 (90.7%)	1.000			
**AFB Smear grade**			0.581			
Negative or 1+	40 (49.4)	19 (44.2)				
2+ or 3+	41 (50.6)	24 (55.8)				
**TB diagnosis**			0.319			
Clinical	10 (12.1%)	8 (18.6%)				
Laboratory confirmed	73 (87.9%)	35 (81.4%)				
**Signs and symptoms**	**Presence**			**Duration**		
	**Adults**	**Elderly**		**Adults**	**Elderly**	
	**n (%)**	**n (%)**	**p value**	**Days**	**Days**	**p value**
**Cough**	79 (95.2%)	39 (92.9%)	0.687	70 (70)	90 (105)	**0.071**
**Productive Cough**	68 (85%)	35 (81.4%)	0.616	30 (40)	60 (70)	0.610
**Hemoptysis**	28 (35%)	13 (31%)	0.691	0 (3)	0 (1.5)	0.904
**Fever/ Chills**	67 (80.7%)	25 (58.1%)	**0.007**	7 (18)	7 (20)	0.612
**Chest Pain**	43 (51.8%)	21 (48.8%)	0.706	14 (30)	15 (30)	0.419
**Weight Loss**	59 (73.8%)	34 (79.1%)	0.660	30 (40)	60 (105)	**0.015**

NAP, Not applicable; p values ≤ 0.099 shown in bold

### Characteristics of the elderly with LTBI

Among the elderly CoC group, more than half had LTBI (Table 1), which puts them at higher risk for TB reactivation when compared to non-infected elderly [4]. However, we did not find any host factor associated with LTBI status among elderly CoC. Furthermore, among elderly ReC there were few differences when comparing positive *vs*. negative LTBI (S5 Table).

## Discussion

We conducted a cross-sectional study to identify unique aspects of TB and LTBI in elderly Hispanics from the Texas-Mexico border region. TB risk factors such as HIV/AIDS, excess alcohol use and drug abuse were more prevalent in adults, and were excluded from analysis. We found that few host factors were associated with LTBI status. In contrast, the elderly had a higher prevalence of birth cohort characteristics that are known risk factors for active TB in adults, such as diabetes and lower education [31, 32]. Interestingly, despite the high prevalence of diabetes in the elderly population, we found that it was not associated with TB. Instead, being male, smoking and having a low BMI were risk factors for TB in the elderly. An additional unexpected finding was the protective effect of BCG vaccination at birth in the elderly.

Our finding that diabetes is not a risk factor for TB in the elderly contrasts with numerous studies in adults, including our own, where diabetes patients have a 1.5 to 3-fold higher risk of TB [15, 17, 33]. We confirmed that adult diabetes patients have a higher risk of TB, and we further observed a higher risk of TB in adults with impaired fasting glucose. However, hyperglycemia and impaired fasting glucose were not associated with TB in the elderly. In fact, impaired fasting glucose was protective by univariable analysis. A reduced strength in the association between TB and diabetes has been noted in past studies, but its implications for the elderly population have not been explicitly evaluated in multivariable models [14, 15]. In fact, diabetes continues to be regarded as a risk factor for TB among the elderly [34, 35]. We speculate that in the elderly, the lack of an association between type 2 diabetes and TB is explained by differences in the underlying pathophysiology of diabetes. Type 2 diabetes in the elderly is characterized by a milder hyperglycemia due to delayed responsiveness of pancreatic beta cells to release insulin *versus* high levels of insulin, insulin resistance and a more severe hyperglycemia in adults. We also cannot rule out a survivor effect of well-controlled diabetes in our elderly cohorts. We are currently evaluating these distinctions.

In experimental models, BCG vaccine can extend the survival of diabetic mice infected with *M*.*tb* through reduced immunopathology, potentially mediated by Th2/M2-mediated mechanisms [36]. Although these studies only evaluated mice for one year, equivalent to mid-life for mice [37], we can perhaps extrapolate that BCG would also afford older diabetic mice protection from TB. Specific studies to address BCG and diabetes in the context of TB have not been performed, but BCG vaccination of old mice [38] and guinea pigs [39] does provide protection against TB. Our findings raise the need to evaluate the occurrence of dysglycemia in old mice, and document its relationship to TB risk. Despite the lack of association between diabetes and TB in the elderly population, we recommend the screening for diabetes in all newly diagnosed elderly TB patients given that management of acute hyperglycemia is important for proper immune function against *M*. *tuberculosis* [40–42]. Furthermore, diabetes may be associated with adverse TB treatment outcomes in the elderly, as is the case for adults [5, 43].

Our findings showed a lack of association between BCG vaccination and TB in adults, which is consistent with the literature [44]. However, a novel observation in our study was that the elderly with history of BCG vaccination were less likely to have TB. BCG may confer protection against LTBI in adults [45], but is mostly known for its protective effect against disseminated TB in children [46]. We are not aware of any other documentation that BCG vaccination at birth will confer protection from TB in elders. A recent meta-analysis concluded that BCG confers protection against TB shortly after vaccination, and hence, its effect is most notable in children but not in adults in high burden settings. Their results in adults do not support our findings, but there is limited data in individuals beyond 60 years of age [47]. Confirmation of our findings in other elderly cohorts would open the possibilities for considering a booster BCG in the elderly. Indeed, BCG induces a non-specific protective effect against other microbial infections in young children through the induction of long-lasting epigenetic changes in the bone marrow myeloid precursor for monocytes, a process known as trained immunity [48]. Evidence for BCG-induced trained immunity in elderly individuals was recently demonstrated by the ACTIVATE trial, in which BCG vaccination of individuals 65 years and older lowered the risk of viral respiratory infections [49], although preliminary analysis of the BCG-PRIME study shows no protection against COVID-19 [50]. We speculate that BCG-induced trained immunity at birth confers a “baseline” protection against active TB development that persists throughout life. This “baseline” protection is most notable when the immune system is still immature in newborns, and when it wanes in the elderly, but not in other age groups where other aspects of the immune system, including adaptive immunity, play a prominent role in *M*.*tb* infection control. Consistent with this possibility, BCG at birth can confer heterologous protection against other respiratory infections, potentially further shaping the pulmonary immune system through adult and elderly life [51].

TB delayed diagnosis or misdiagnosis and high mortality rates in the elderly are attributed to their increased likelihood of absent, altered, or delayed clinical symptoms, presence of age-associated conditions such as cognitive impairment, and clinical symptoms shared by active TB and old age such as fatigue, and weight loss [5, 30, 52, 53]. In our elderly cohort, we found clinical differences such as a lower prevalence of fever and chills, and longer duration of cough and weight loss. In fact, several elderly TB patients died prior to enrollment in our study or TB treatment initiation. There is a need for biomarkers to enhance TB diagnosis and prognosis in this population. Candidates include the higher neutrophil/lymphocyte and monocyte/lymphocyte ratios observed by others and us, associated with more severe TB in adults [54–59].

Study limitations are the small sample size of our elderly cohort, particularly for some sub-analysis. We also defined elderly participants as those 60 years and older, but TB risk increases with age among the elderly, with waning representation in the oldest age groups [4]. Our study could be affected by survival bias, with some TB patients dying prior to enrollment.

In summary, our findings in elderly Hispanics confirm the presence of shared risk factors for TB with adults, such as being male, smoking and low BMI. Importantly, our findings highlight differences between age groups, notably the lack of an association between TB and diabetes, and the potential protective effect of BCG vaccination in the elderly population. Despite its low prevalence in our study population, smoking was another independent risk factor for TB that deserves further evaluation in other elderly cohorts. Our results call for future studies in the elderly population, for tailored identification of risk factors for LTBI, active TB or adverse TB outcomes, in this unique, heterogeneous, vulnerable and understudied population.

## Supporting information

S1 Fig Sociodemographic and health-related conditions by age group and TB status. Percentage of adults (A) and elderly (E) with TB and without TB (No TB) for select sociodemographic factors and health-related conditions. For smoking history, A and E with TB and No TB are shown as smoking pack per year. Student’s t test between age groups (A vs E) among participants with No TB or TB; *p ≤ 0.05. Dotted lines for BMI indicate cut-offs for underweight (<18.5), normal (18.5–24.9), overweight (25–30) and obese (<30); LTBI, latent TB infection; NSAIDs, nonsteroidal anti-inflammatory drugs.
(DOCX)

S2 Fig Lipid and complete blood count and differentials by age group and TB status. Percentage of adults (A) and elderly (E) with TB and without TB (No TB) with high cholesterol, high LDL, low HDL, and high triglycerides (*top row*). Bottom row shows complete blood counts (x1e3/μL) for immune cell populations and neutrophil: Lymphocyte and monocyte: Lymphocyte ratios by age and TB status (TB and No TB). Student’s t test between age groups (A vs E) among participants with No TB or TB; *p ≤ 0.05; # p between 0.051–0.099.
(DOCX)

S3 Fig Distinguishing characteristics of elderly with TB versus no TB. Percentage of EL with TB and without TB (No TB) for select sociodemographic factors, health-related conditions, and lipids. Student’s t test between elderly (EL) with No TB and EL with TB; *p ≤ 0.05. IFG, Impaired fasting glucose.
(DOCX)

S1 Table Characteristics of the elderly *vs*. adults, among CoC or ReC groups. (DOCX)

S2 Table Complete blood counts and differential in elderly *vs*. adults, by TB status. (DOCX)

S3 Table Characteristics of TB *vs*. non-TB patients in the elderly. (DOCX)

S4 Table Host factors associated with BCG vaccination among all study participants. (DOCX)

S5 Table Unique characteristics in the elderly recent TB contacts, by LTBI status. (DOCX)
