# Supplementary figures and images for: A prospective cross-sectional study of tuberculosis in elderly Hispanics reveals that BCG vaccination at birth is protective whereas diabetes is not a risk factor
Source: PLoS One. 2021 Jul 29;16(7):e0255194. doi: 10.1371/journal.pone.0255194 (PMC8321126; doi:10.1371/journal.pone.0255194)

**S1 Fig.**

**
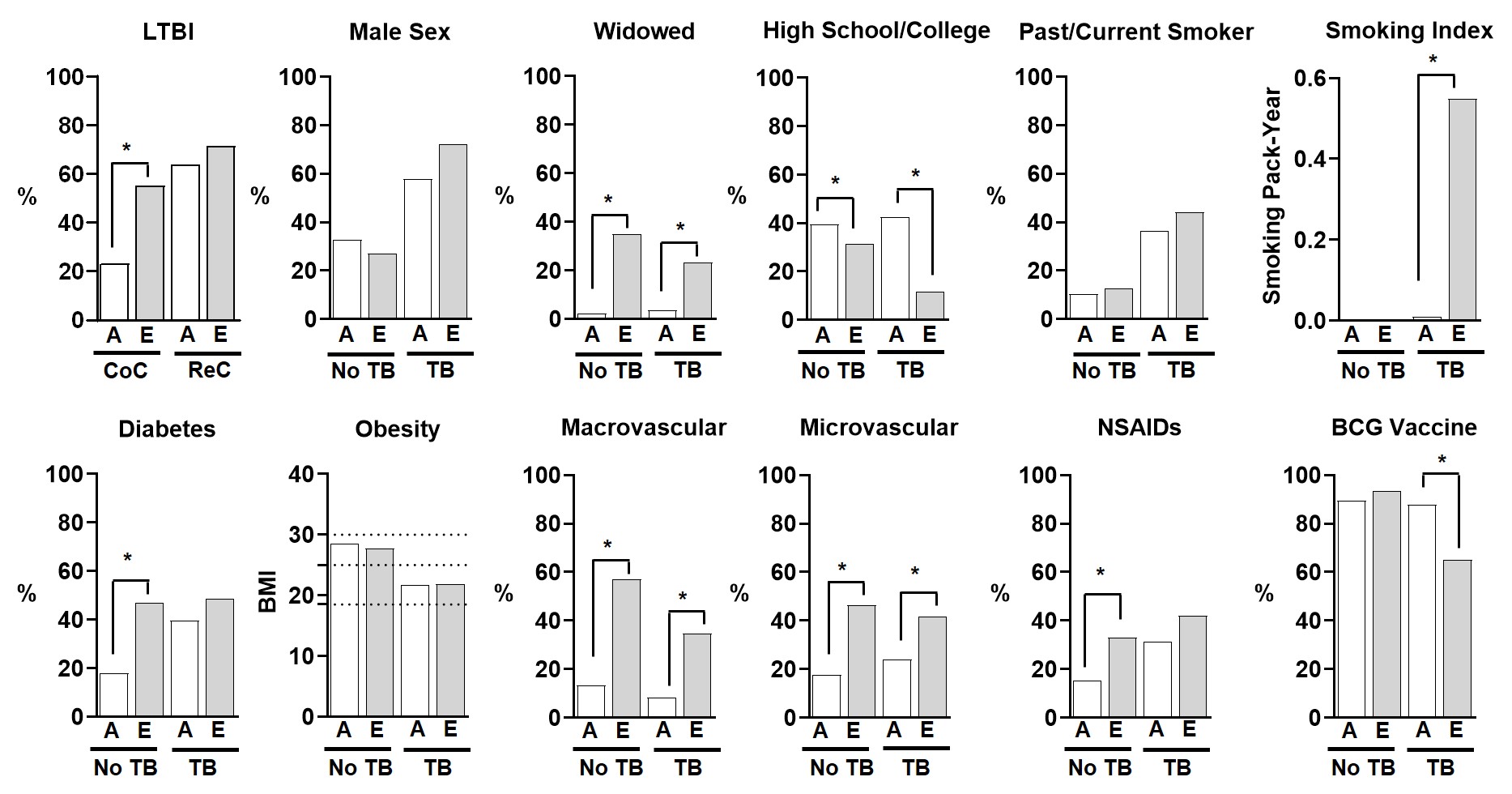
**

Supplement: S1 Fig — Percentage of adults (A) and elderly (E) with TB and without TB (No TB) for select sociodemographic factors and health-related conditions. For smoking history, A and E with TB and No TB are shown as smoking pack per year. Student’s t test between age groups (A vs E) among participants with No TB or TB; *p ≤ 0.05. Dotted lines for BMI indicate cut-offs for underweight (<18.5), normal (18.5–24.9), overweight (25–30) and obese (<30); LTBI, latent TB infection; NSAIDs, nonsteroidal anti-inflammatory drugs. (DOCX) [file pone.0255194.s001.docx]

**S2 Fig.**


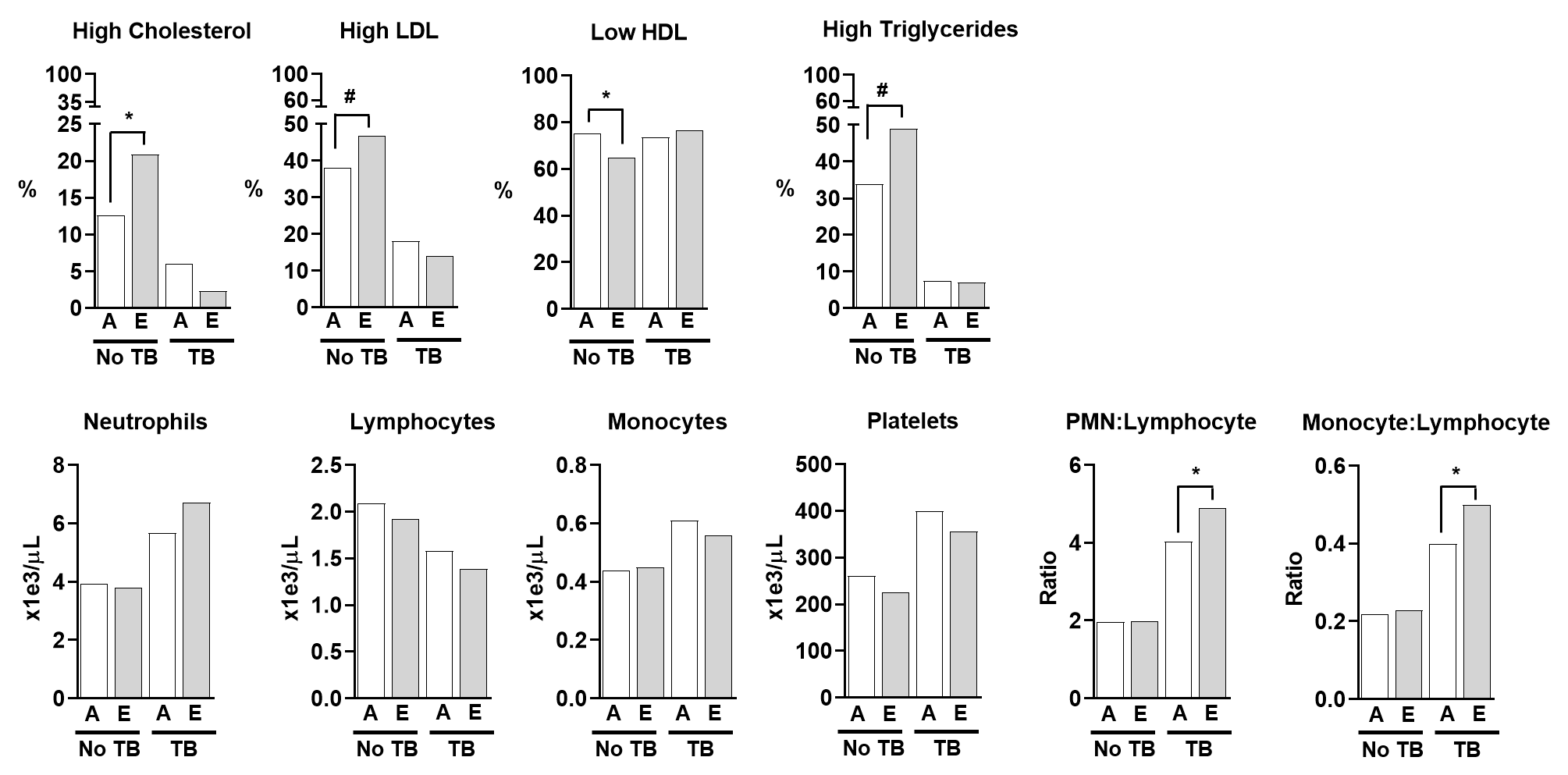

Supplement: S2 Fig — Percentage of adults (A) and elderly (E) with TB and without TB (No TB) with high cholesterol, high LDL, low HDL, and high triglycerides (top row). Bottom row shows complete blood counts (x1e3/μL) for immune cell populations and neutrophil: Lymphocyte and monocyte: Lymphocyte ratios by age and TB status (TB and No TB). Student’s t test between age groups (A vs E) among participants with No TB or TB; *p ≤ 0.05; # p between 0.051–0.099. (DOCX) [file pone.0255194.s002.docx]
