# Supplementary material for: A prospective cross-sectional study of tuberculosis in elderly Hispanics reveals that BCG vaccination at birth is protective whereas diabetes is not a risk factor
Source: PLoS One. 2021 Jul 29;16(7):e0255194. doi: 10.1371/journal.pone.0255194 (PMC8321126; doi:10.1371/journal.pone.0255194)
Supplement: S1 Table — (DOCX) [file pone.0255194.s004.docx]

**Supporting material**

**RESULTS**

**Age-associated characteristics between elderly and adults within community controls (CoC) or recent contacts (ReC)**

Among the CoC the following features were more prevalent in the elderly (E) *vs.* adults (A) (p≤0.099; **S1 Table** below). In socio-demographics the elderly had a higher prevalence of females (E 81.7% *vs.* A 59.8%), and differences in socioeconomic measures [lower education (E 28.3% *vs.* A 70.7%), but higher health insurance (E 95.0% *vs.* A 75.3%)]. In health conditions, the elderly had a higher prevalence of diabetes (E 43.3% *vs.* A 19.5%), pre-diabetes (E 36.7% *vs.* A 22.0%), BCG vaccination history (E 98.3% *vs*. A 89.0% in adults), LTBI (E 55.0% *vs.* A 23.2%; **Fig. S1**), macrovascular (E 71.7% *vs.* A 12.2%) or microvascular diseases (E 48.3% *vs.* A 19.5%), and the use of anti-inflammatory medications.

Among the 381 ReC (80 elderly and 301 adults), the following features were more prevalent in the elderly (p<0.099; **S1 Table**). In socio-demographics widowed status was more prevalent (E 30.0% *vs*. A 2.0%) and smoking pack-years was higher. In socioeconomics there were fewer individuals per household [median (IQR), E 3 (3) *vs.* A 4 (3)]. Regarding health conditions, the following conditions were more prevalent: Diabetes (E 50.0% *vs.* A 17.6%), pre-diabetes (E 25.0 *vs.* A 20.9%), macrovascular (E 46.3% *vs.* A 13.6%) and microvascular diseases (E 45.0% *vs.* A 17.3 %), and the use of anti-inflammatory medications.
