# Supplementary material for: A prospective cross-sectional study of tuberculosis in elderly Hispanics reveals that BCG vaccination at birth is protective whereas diabetes is not a risk factor
Source: PLoS One. 2021 Jul 29;16(7):e0255194. doi: 10.1371/journal.pone.0255194 (PMC8321126; doi:10.1371/journal.pone.0255194)
Supplement: S2 Table — (DOCX) [file pone.0255194.s005.docx]

| **S2 Table. Complete blood counts and differential in elderly *vs.* adults, by TB status** | | | | | | | | | | | | | | | |
| --- | --- | --- | --- | --- | --- | --- | --- | --- | --- | --- | --- | --- | --- | --- | --- |
|  | **Non-TB** | | | | | | |  | **TB** | | | | | | |
|  | **Adults** | |  | **Elderly** | |  |  |  | **Adults** | |  | **Elderly** | |  |  |
|  | **n** | **Med (IQR)** |  | **n** | **Med (IQR)** |  | **p value** |  | **n** | **Med (IQR)** |  | **n** | **Med (IQR)** |  | **p value** |
| **Absolute values** (x1e3/µL for cells or platelets) | | | | | | | | | | | | | | | |
| **White blood cells** (4.8-10.9) | 314 | 6.85 (2.3) |  | 128 | 6.7 (2.05) |  | 0.147 |  | 64 | 8.50 (4.35) |  | 41 | 8.90 (3.70) |  | 0.340 |
| **Neutrophils** (2.3-7.7) | 314 | 3.94 (1.77) |  | 128 | 3.79 (1.57) |  | 0.139 |  | 63 | 5.68 (3.48) |  | 41 | 6.72 (3.45) |  | 0.250 |
| **Lymphocytes** (0.8-3.3) | 314 | 2.09 (0.71) |  | 128 | 1.92 (0.7) |  | 0.104 |  | 63 | 1.58 (0.71) |  | 41 | 1.39 (1.07) |  | 0.270 |
| **Monocytes** (0.2-1.0) | 313 | 0.44 (0.16) |  | 128 | 0.45 (0.16) |  | 0.930 |  | 63 | 0.61 (0.40) |  | 41 | 0.56 (0.33) |  | 0.992 |
| **Eosinophils** (0.0-0.4) | 314 | 0.16 (0.16) |  | 128 | 0.18 (0.2) |  | **0.045** |  | 63 | 0.15 (0.17) |  | 41 | 0.16 (0.20) |  | 0.981 |
| **Platelets** (146-388) | 291 | 261 (78) |  | 124 | 225 (79) |  | **<0.001** |  | 64 | 399.00 (194.00) |  | 41 | 356.00 (168.00) |  | 0.347 |
| **Hemoglobin** (11.6-15.9 mg/dL) | 291 | 13.4 (2.3) |  | 124 | 13.1 (1.8) |  | **0.047** |  | 64 | 12.20 (2.70) |  | 41 | 11.90 (2.00) |  | 0.400 |
| **Ratios** | | | | | | | | | | | | | | | |
| **Monocyte:lymphocyte** | 314 | 0.22 (0.09) |  | 128 | 0.23 (0.1) |  | 0.210 |  | 63 | 0.40 (0.24) |  | 41 | 0.50 (0.31) |  | **0.029** |
| **Neutrophil:lymphocyte** | 314 | 1.96 (1) |  | 128 | 1.98 (1) |  | 0.962 |  | 63 | 4.04 (2.69) |  | 41 | 4.90 (5.78) |  | **0.045** |
| * Normal range values for each parameter shown in parentheses; p values ≤ 0.099 shown in bold; Difference in median values between groups were compared using the Kruskal-Wallis test with Dunn's multiple comparisons post-test. | | | | | | | | | | | | | | | |
