# Supplementary material for: A prospective cross-sectional study of tuberculosis in elderly Hispanics reveals that BCG vaccination at birth is protective whereas diabetes is not a risk factor
Source: PLoS One. 2021 Jul 29;16(7):e0255194. doi: 10.1371/journal.pone.0255194 (PMC8321126; doi:10.1371/journal.pone.0255194)
Supplement: S3 Table — (DOCX) [file pone.0255194.s006.docx]

| **S3 Table. Characteristics of TB *vs.* non-TB patients in the elderly** | | | | | | | |
| --- | --- | --- | --- | --- | --- | --- | --- |
|  | **Non-TB** | |  | **TB** | |  |  |
|  | **n** | **% or  median (IQR)** |  | **n** | **% or  median (IQR)** |  | **p value** |
| **Sociodemographics** | | | | | | | |
| **Male sex** | 38 | 27.1% |  | 31 | 72.1% |  | **<0.001** |
| **Highest education** |  |  |  |  |  |  | 0.248 |
| Up to Middle School | 113 | 80.7% |  | 38 | 88.4% |  |  |
| High School or College | 27 | 19.3% |  | 5 | 11.6% |  |  |
| **Smoking, Current or past** | 35 | 24.8% |  | 23 | 54.5% |  | **< 0.001** |
| **Diabetes and related conditions** | | | | | | | |
| **Diabetes** | 66 | 47.1% |  | 21 | 48.8% |  | 0.846 |
| **Diabetes and pre-diabetes** |  |  |  |  |  |  | 0.584 |
| No | 33 | 23.4% |  | 7 | 16.3% |  |  |
| Pre-diabetes | 42 | 29.8% |  | 15 | 34.9% |  |  |
| Diabetes | 66 | 46.8% |  | 21 | 48.8% |  |  |
| **Glycemia** |  |  |  |  |  |  | **0.058** |
| Normoglycemia | 78 | 55.7% |  | 32 | 74.4% |  |  |
| Impaired Fasting Glucose | 22 | 15.7% |  | 2 | 4.7% |  |  |
| Hyperglycemia | 40 | 28.6% |  | 9 | 20.9% |  |  |
| **HbA1c** |  |  |  |  |  |  | 0.614 |
| <5.7% | 36 | 25.7% |  | 8 | 18.6% |  |  |
| 5.7-6.49% | 56 | 40.0% |  | 18 | 41.9% |  |  |
| >6.49% | 48 | 34.3% |  | 17 | 39.5% |  |  |
| **Obesity and lipid profiles** | | | | | | | |
| **Obesity, BMI** |  |  |  |  |  |  | **<0.001** |
| Underweight/normal | 40 | 28.4% |  | 36 | 83.7% |  |  |
| Overweight/Obese | 101 | 71.6% |  | 7 | 16.3% |  |  |
| **Central obesity, waist-hip ratio** |  |  |  |  |  |  | **<0.001** |
| Normal | 28 | 20.0% |  | 20 | 46.5% |  |  |
| High | 111 | 79.3% |  | 22 | 51.2% |  |  |
| **Lipid profiles**, (mg/dl) |  |  |  |  |  |  |  |
| **High cholesterol** (200) | 29 | 20.7% |  | 1 | 2.3% |  | **0.004** |
| **Low HDL** (40 M, 50 F) | 90 | 64.3% |  | 33 | 76.7% |  | 0.149 |
| **High LDL** (100) | 65 | 46.4% |  | 6 | 14.0% |  | **<0.001** |
| **High Triglycerides** (150) | 46 | 32.9% |  | 3 | 7.0% |  | **<0.001** |
| **Other health-related conditions** | | | | | | | |
| **BCG vaccine** | 131 | 93.6% |  | 28 | 65.1% |  | **<0.001** |
| **Macrovascular diseases** | 80 | 57.1% |  | 15 | 34.9% |  | **0.011** |
| **Microvascular diseases** | 65 | 46.4% |  | 18 | 41.9% |  | 0.599 |
| Normal range values for each parameter shown in parenthesis; M, male; F, female; p values ≤ 0.099 shown in bold; | | | | | | | |
