# Supplementary material for: A prospective cross-sectional study of tuberculosis in elderly Hispanics reveals that BCG vaccination at birth is protective whereas diabetes is not a risk factor
Source: PLoS One. 2021 Jul 29;16(7):e0255194. doi: 10.1371/journal.pone.0255194 (PMC8321126; doi:10.1371/journal.pone.0255194)
Supplement: S4 Table — (DOCX) [file pone.0255194.s007.docx]

| **S4 Table. Host factors associated with BCG vaccination among all study participants** | | | | | | | | |
| --- | --- | --- | --- | --- | --- | --- | --- | --- |
|  |  | **No BCG** | |  | **Yes BCG** | |  |  |
|  |  | **n** | **%** |  | **n** | **%** | **p value** | **Adj p value** |
| **Age Groups** |  |  |  |  |  |  | 0.416 |  |
| 18-50y |  | 50 | 10.90% |  | 411 | 89.10% |  |  |
| 60+y |  | 24 | 13.10% |  | 159 | 86.90% |  |  |
| **Sex** |  |  |  |  |  |  | **0.014** |  |
| Female |  | 37 | 9.1% |  | 369 | 90.9% |  |  |
| Male |  | 37 | 15.6% |  | 201 | 84.4% |  |  |
| **TB** |  |  |  |  |  |  | **<0.001** |  |
| No |  | 49 | 9.4% |  | 472 | 90.6% |  |  |
| Yes |  | 25 | 20.3% |  | 98 | 79.7% |  |  |
| **Education** |  |  |  |  |  |  | **0.027** | **0.045** |
| Up to middle school |  | 54 | 13.7% |  | 340 | 86.3% |  |  |
| High School or higher |  | 20 | 8.0% |  | 230 | 92.0% |  |  |
| **Health Insurance** |  |  |  |  |  |  | **0.027** | **0.025** |
| No |  | 26 | 16.2% |  | 135 | 83.8% |  |  |
| Yes |  | 47 | 9.8% |  | 434 | 90.2% |  |  |
| Only variables associated with BCG vaccination are shown; Discrete variables shown as **row %**; p value adjusted for age, sex and TB status | | | | | | | | |
