# Supplementary material for: A prospective cross-sectional study of tuberculosis in elderly Hispanics reveals that BCG vaccination at birth is protective whereas diabetes is not a risk factor
Source: PLoS One. 2021 Jul 29;16(7):e0255194. doi: 10.1371/journal.pone.0255194 (PMC8321126; doi:10.1371/journal.pone.0255194)
Supplement: S5 Table — (DOCX) [file pone.0255194.s008.docx]

| **S5 Table. Unique characteristics in the elderly recent TB contacts, by LTBI status *** | | | | | | | | |
| --- | --- | --- | --- | --- | --- | --- | --- | --- |
|  | **Latent TB infection** | | | | |  | **Latent TB infection Yes/No** | |
|  | **No** | |  | **Yes** | |  |  |  |
|  | **n** | **% or  median (IQR)** |  | **n** | **% or  median (IQR)** |  | **Crude OR or β  (95% CI)** | **Adj β or OR * (95% CI)** |
| **Macrovascular disease** | 6 | 26.1% |  | 31 | 54.4% |  | **3.38 (1.16, 9.82)** | **3.40 (1.13, 10.26)** |
| **High LDL** (100 mg/dL) | 14 | 60.9% |  | 24 | 42.1% |  | **0.42 (0.15, 1.15)** | **0.39 (0.14, 1.10)** |
| **Neutrophils** (2.3-7.7x1e3/ul) | 20 | 4.24 (2.25) |  | 51 | 3.81 (1.68) |  | **-0.72 (-1.41, -0.02)** | **-0.71 (-1.37, -0.05)** |
| **Platelets** (146-388x1e3/ul) | 20 | 273.00 (93.50) |  | 47 | 211.00 (65.00) |  | **-53.02 (-86.16, -19.88)** | **-51.44 (-83.58, -19.34)** |
| *Only variables with p lt 0.09 are shown; Normal range values for each parameter shown in parenthesis; ** Adjusted for sex and age. β, beta coefficient; 95% CI with p values ≤ 0.099 shown in bold; | | | | | | | | |
